# FAM134B-mediated endoplasmic reticulum autophagy protects against sepsis myocardial injury in mice

**DOI:** 10.18632/aging.202786

**Published:** 2021-03-26

**Authors:** Tong Li, Yongsheng Chen, Yue Li, Zhipeng Yao, Wenhua Liu

**Affiliations:** 1 Intensive Care Unit, The Second Affiliated Hospital of Harbin Medical University, Harbin, PR China; 2 Department of Urology, Harbin Medical University Cancer Hospital, Harbin, PR China

**Keywords:** reticulophagy regulator 1, endoplasmic reticulum autophagy, inflammation, apoptosis, LC3-II/I

## Abstract

Reticulophagy regulator 1 (RETEG1, also known as FAM134B) plays a crucial role in endoplasmic reticulum autophagy. We aimed to explore the effect of FAM134B-mediated endoplasmic reticulum autophagy in sepsis myocardial injury in mice. Sepsis myocardial injury mice were established via cecal ligation and puncture procedures. The expression of FAM134B and LC3-II/I was determined using immunohistochemistry. Myocardial tissue morphological changes and apoptosis were examined using hematoxylin and eosin (H&E) staining and TUNEL analysis. The effects of FAM134B knockdown or overexpression on mice with sepsis myocardial injury were also studied. The levels of TNF-α, IL-6, IL-8, and IL-10 were evaluated using enzyme-linked immunosorbent assay (ELISA). Autophagy- and apoptosis-related protein expression was detected using western blotting. The effect of FAM134B on Lipopolysaccharide (LPS) -induced cardiomyocytes was also studied. The expression of FAM134B and LC3-II/I increased in sepsis mice and lipopolysaccharide (LPS)-treated cardiomyocytes. 3-Methyladenine (3-MA) significantly inhibited FAM134B and LC3-II/I expression and promoted myocardial injury, inflammation response, and cardiomyocyte apoptosis. The overexpression of FAM134B could minimize myocardial injury, inflammation, and apoptosis, whereas FAM134B knockdown showed opposite effects. FAM134B-mediated endoplasmic reticulum autophagy had a protective effect on sepsis myocardial injury in mice by reducing inflammation and tissue apoptosis, which may provide new insights for sepsis myocardial injury therapies.

## INTRODUCTION

Sepsis is a life-threatening multi-organ dysfunction caused by an imbalance in the body's response to severe infections [1, 2]. It is the first fatal factor in critically ill patients, with a mortality rate higher than 50%. Cardiac dysfunction is an important component of multiple organ dysfunction in sepsis [3, 4]. Approximately 40–50% of sepsis patients have complications associated with cardiac dysfunction. After diagnosis and treatment, although the cardiac output increased and the cardiac output returned to normal, the myocardial dysfunction was still very obvious. Vascular leakage caused by inflammation can lead to myocardial edema and affect cardiac compliance and function. At present, a dysfunction of myocardial energy metabolism, an abnormal calcium homeostasis, and the apoptosis of myocardial cells are important mechanisms of myocardial dysfunction in sepsis [5, 6].

Endoplasmic reticulum (ER) is the base for synthesis of some important biomacromolecules, such as proteins, lipids and sugars. [7, 8]. When cells are stimulated by internal and external factors, the shape of ER changes and protein processing and transportation are blocked. A large number of unfolded or misfolded proteins accumulate in the ER. In order to survive to ER stress, the ER chaperon protein, glucose-regulated protein (GRP78) and other ER chaperones were activated to show protective effects [9, 10]. FAM134B plays a crucial role ER autophagy. Previous studies have reported that FAM134B is an ER-anchored autophagy receptor that mediates ER delivery into lysosomes through sequestration into autophagosomes ^11^. FAM134B can promote membrane remodeling and ER disruption through its membrane bending ability and target the fragments into autophagosomes via interaction with ATG8 family proteins [11]. In addition, FAM134B is also required for the long-term survival of nociceptive and autonomic ganglion neurons [12].

In the present study, We first found that FAM134B was overexpressed in sepsis mice and lipopolysaccharide (LPS)-treated cardiomyocytes. Our goal was to explore the effect of FAM134B-mediated ER autophagy in sepsis myocardial injury in mice and to demonstrate its underlying mechanism.

## MATERIALS AND METHODS

### Animals

We used male C57BL/6 mice (weight: 20–25 g) for *in vivo* study. The mice were provided by the Experimental Animal Center of The Second Affiliated Hospital of Harbin Medical University. The mice were housed separately and kept under standard conditions at room temperature (22–24° C) under a 12:12 h light/dark cycle. The protocols of the present study were carried out in accordance with the guidelines for animal experiments of the Animal Ethics Committee of the Second Affiliated Hospital of Harbin Medical University. The animal study was also approved by the Animal Ethics Committee of the Second Affiliated Hospital of Harbin Medical University.

### Construction of septic mouse model

Cecal ligation and puncture (CLP) treatment was performed to establish the septic mouse model, as previously reported [13, 14]. Mice were anesthetized with 0.1% pentobarbital sodium. After ligation of exposed cecum, 22G needle was used to puncture twice and part of feces was extruded to ensure smooth puncture. Following this, the bowel loops were returned and the abdominal wall was closed using a 4-0 silk suture. Postoperatively, 1 mL of 0.9% saline was injected subcutaneously. In the sham operation group, mice were exposed to cecum and returned without puncture and ligation. Mice were then injected with 1ml 0.9% normal saline. In the present experiment, the mortality of C57BL/6 mice at 72 hours after CLP was about 60-80%. Rapamycin (Rap) was dissolved in dimethyl sulfoxide (DMSO) and was intraperitoneally injected (10 mg/kg) 1 h after CLP [15]. 3-MA was dissolved in ddH_2_O and intraperitoneally injected (10 mg/kg) 1 h after CLP [16]. The plasmids of FAM134B overexpression (oeFAM134B) were constructed by inserting the amplified FAM134B cDNA into the pCDNA3.1 vector (Invitrogen, Shanghai, China). Small interfering RNAs targeting FAM134B (siFAM134B: 5′-GCTCAGCCACTGTATTGCAGAATCA-3′) were designed and synthesized by Shanghai Genechem Co., Ltd. (Shanghai, China), as well as the negative control siRNAs (5′-GCACTCGGAGACTTAAGCACTAACA-3′). Subsequently, tail vein intravenous injections of oeFAM134B at a dose of 30 mg/kg body weight were administered before the CLP operation. FAM134B knockdown was performed via tail vein intravenous injections of siFAM134B at a dose of 80 mg/kg body weight for three consecutive days before the CLP operation. Transfection efficiency was examined using reverse transcription polymerase chain reaction (RT-PCR).

### Histopathological evaluation of the heart tissues

The histopathological changes in the heart tissues were evaluated by hematoxylin and eosin (H&E) staining. We collected heart tissues and stored them at -80° C, and then they were cut into 5 μm sections. The sections were treated with H&E staining kit (Solarbio, Beijing, China) according to the manufacturer’s instructions. The observation of histological changes in heart tissues was assessed by a light microscope (Olympus, Tokyo, Japan).

### TUNEL assay

Cell apoptosis in myocardial tissue was examined by a fluorescence detection kit (Merck-millipore, Berlin, Germany) and a fluorescence microscopy (BD, New York, USA). The myocardial tissue was embedded in paraffin, cut into 5 m thick sections, and then dehydrated. The sections were incubated with protease K (Invitrogen) solution at 25° C for 30 minutes. The TUNEL reaction mixture containing TdT and fluorescein dUTP was added to the sections and incubated in a dark humidified chamber at 37° C for 60 minutes. The slices were washed with PBS for 5 min each time. After implantation, images of these slices were visualized using microscope and the apoptosis rate was assessed by ImageJ software.

### Immunofluorescence assay

The heart tissues of each group were embedded in paraffin and cut into 4 μm sections. Antigen recovery was performed in EDTA buffer (Absin, Shanghai, China) and heated to 99° C for 20 minutes. After quenching the endogenous peroxidase with 3% hydrogen peroxide, it was blocked with 10% non immune goat serum (Invitrogen). The sections were then incubated overnight with anti-FAM134B (1:100, Cat. QM13136R, KA&M-BIO, Shanghai, China) and anti-LC3-II/I (1:100, Cat. ABC929, Millipore) overnight at 4° C. The sections were washed with TBST, and then co-incubated with secondary antibody (GENMED, Shanghai, China) and fluorescein wheat germ agglutinin (Vectorlabs, New York, USA). The nuclei were stained with DAPI (Invitrogen). The images were taken with confocal microscope (CSIM100, SUNNY, Beijing, China).

### RNA extraction and RT-PCR analysis

Total RNA from cells or tissues was extracted by using TRIzol (Balb, Beijing, China). Reverse transcription into cDNA was performed using 200 ng of total RNA and cDNA reverse transcription Kit (MultiSciences, Hangzhou, China) according to the manufacturer’s instructions, and the cDNAs were stored at -80° C. Semi-quantitative RT-PCR was employed to detect the expressions of *Caspase-3* (F: 5’-CCACAGCACCTGGTTATT-3’, R: 5’-ATTCTATCGCCACCTTCC-3’), *Bax* (F: 5’-CCAGGATGCGTCCACCAA-3’, R: 5’-AAAGTAGAAGAGGGCAACCAC-3’), *Bcl-2* (F: 5’-GTGGCCTTCTTTGAGTTCG-3’, R: 5’-ACCCAGCCTCCGTTATCC-3’), *LC3-II* (F: 5’-ACAGTTGGCACAAACGC-3’, R: 5’-CCCTGCAAGAGTGAGGAC-3’), *IRE1α* (F: 5’-GCAGCTCCAGTTCTTCCAG-3’, R: 5’-GCCAGTCCATCTTCACCAC-3’), *GRP78* (F: 5’-AGGAGGACAAGAAGGAGGA-3’, R: 5’-GAGTGAAGGCGACATAGGA-3’), *Beclin-1* (F: 5’-CAGCCGAAGACTGAAGGT-3’, R: 5’-CGTTGAGCTGAGTGTCCA-3’), and *LAMP2* (F: 5’-CAACCCCAATACAACTCACTC-3’, R: 5’-ATGCTGATGTTCACTTCCTTC-3’). The RT-PCR reactions were incubated in 96-well optical plates at 95° C for 5 min, followed by 40 cycles at 95° C for 20 s, 60° C for 40 s, and 72° C for 8 min. The threshold cycle (Ct) is used calculate the mRNA expressions (2-ΔΔCT). Reactions were run in duplicates to triplicates per RNA isolate. The primer sequences were designed by Sangon Biotech (Shanghai, China). *GAPDH* (F: 5’-AAGGTCGGAGTCAACGGA-3’, R: 5’-TTAAAAGCAGCCCTGGTGA-3’) was used as an internal control.

### Western blotting

In short, 50 μg total protein was subjected to SDS-PAGE and transferred to PVDF membrane. The membrane was sealed with 5% skimmed milk at room temperature of 25° C for 3 hours and incubated with primary antibodies (Abcam, Cambridge, UK) specific to FAM134B (1:1,000, ab151755), cleaved-caspase-3 (1:500, ab49822), caspase-3 (1:500, ab13847), Bcl-2 (1:800, ab196495), Bax (1:800, ab32503), LC3-I/II (1:1,000, ab51520), IRE1α (1:1,000, ab48187), GRP78 (1:1,000, ab21685), Beclin-1 (1:1,000, ab207612), LAMP2 (1:1,000, ab203224), and GAPDH (1:1,000, ab181602) at 4° C overnight. On the next day, membranes were incubated with HRP-conjugated secondary antibodies diluted at 1:3,000 (Boster, Wuhan, China) at 37° C for 1 h. ECL Kit (Agrisera, Sweden) and FluorChem FC3 system (Cell Biosciences, USA) were used to observe the protein bands on the membrane. The results were exhibited as the density ratio between proteins and the load control (GAPDH).

### Cell culture and treatment

The cardiomyocytes were isolated from the left ventricles of mice, as previously described [17], and then cultured in myocyte growth medium (Cat. C-22170, PromoCell, Berlin, Germany) under an atmosphere of 5% CO_2_ and 95% air at 37° C. The plasmids for FAM134B overexpression or siRNAs for FAM134B knockdown were constructed by Shanghai Genechem Co., Ltd. (Shanghai, China). For cell transfection, according to the manufacturer’s instructions, plasmids or siRNAs were transfected into cardiomyocytes by using Lipofectamine 2000 (Invitrogen, Thermo Fisher Scientific, USA), respectively. Transfected cardiomyocytes were cultured for 48 hours, prior to LPS, LPS + Rap (50 nM), or LPS + 3-MA (5 mM) treatment. Following this, the cardiomyocytes were incubated with 0.5 μg/mL LPS or saline solution for 12 h. After that, cardiomyocytes were washed and collected for further experiments.

### Flow cytometry

After 48 hours of LPS treatment, cardiomyocytes were washed with pre-cooled PBS twice, and then resuspended in 400 μL of 1× binding buffer. The same amount (5 μL) of FITC Annexin V and propidium iodide (PI) were lightly mixed into the cell suspension. Cell apoptosis was detected by flow cytometry after 15 minutes of dark culture at room temperature of 25° C.

### ELISA

The myocardial tissue homogenate was prepared by grinding on ice, and the homogenate was centrifuged at 3,000 rpm for 10 min to obtain the supernatant. Cardiomyocytes were harvested and washed three times with PBS. After centrifugation for 5 min at 1,000 rpm, cardiomyocytes were incubated with PBS containing cell lysates for 10 min at 4° C. The supernatant was harvested after centrifugation for 5 min at 12,000 rpm. Inflammatory factors (TNF-α, IL-6, IL-8, and IL-10) content in these supernatant samples was determined using an ELISA kit (Cusabio Biotech, Newark, USA).

### Statistical analysis

SPSS 22.0 was employed for data analysis, and data from at least three independent experiments were presented as mean ± SD. Statistical analysis was carried out using Student's t-test or one-way analysis of variance. A *P* < 0.05 indicated statistical significance.

## RESULTS

### FAM134B expression increased in mice with sepsis myocardial injury

**Figure 1:**
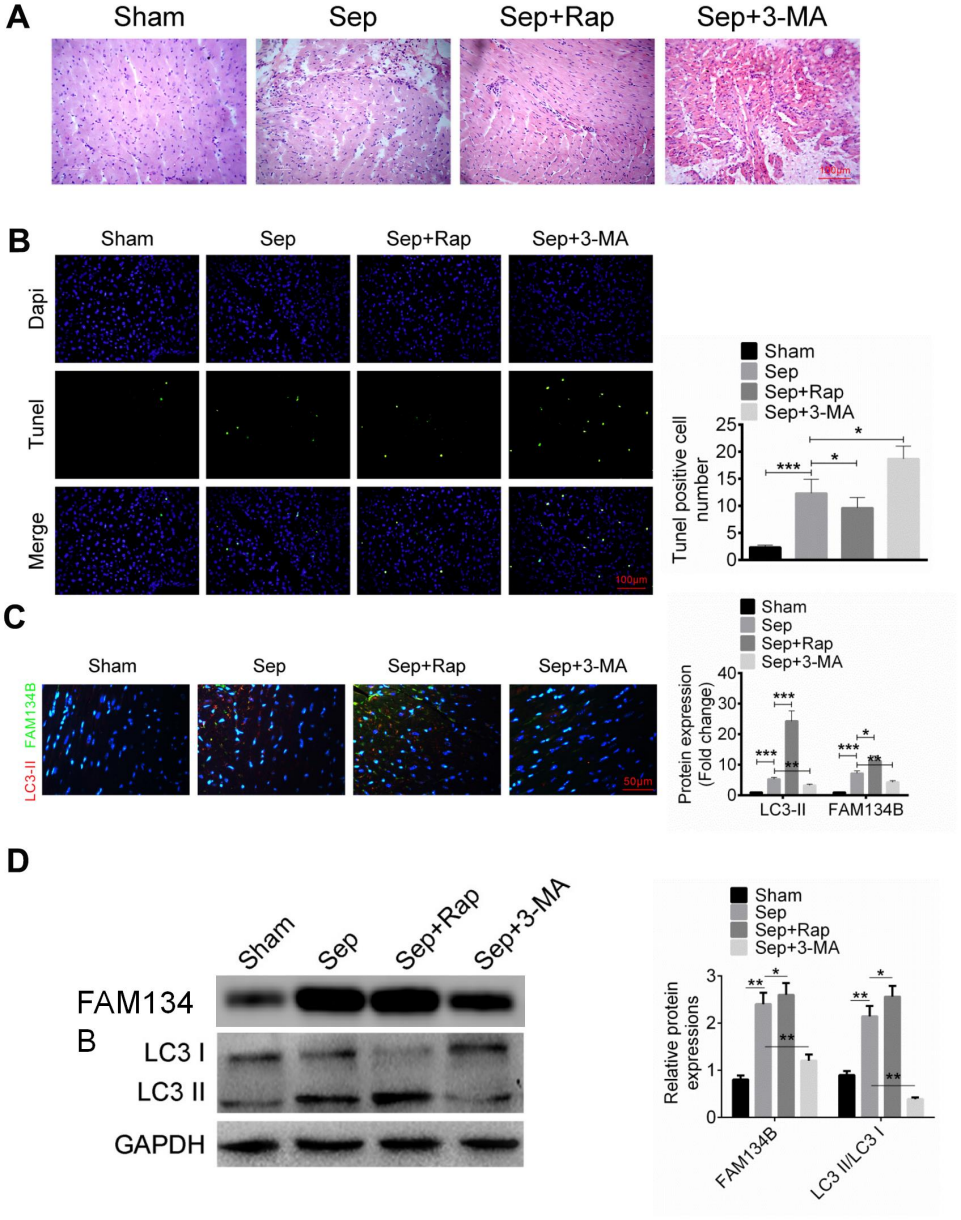
**The expression of FAM134B in the mice with sepsis myocardial injury.** (**A**) Mice were divided into four groups: Sham, Sep, Sep + Rap group, and Sep + 3-MA group, and the myocardial histomorphology was examined by H&E staining. (**B**) Myocardial apoptosis was identified by TUNEL analysis. (**C**, **D**) Protein expressions of LC3-II/I (red fluorescence) and FAM134B (green fluorescence) in the myocardial tissues were examined by immunofluorescence and western blot. Data are shown as mean ± SD. *P<0.05, **P<0.01 and ***P<0.001. Sep: Sepsis. SD: standard deviation.

To identify the biological role of FAM134B in sepsis myocardial injury, mice were treated with cecal ligation and puncture to establish a sepsis myocardial injury model. As shown in Figure 1A, sepsis significantly induced myocardial tissue injury in mice compared with the sham group, using H&E staining. Rap, a known autophagy inducer, effectively decreased the damage induced by sepsis in the myocardial tissue (Figure 1A). Moreover, 3-MA is an autophagy inhibitor that aggravates myocardial tissue injury in mice. Apoptosis in myocardial tissue was examined using TUNEL analysis, and the results revealed that sepsis significantly induced myocardial tissue apoptosis in mice compared with the sham group (Figure 1B). 3-MA treatment enhanced apoptosis and Rap treatment attenuated apoptosis in mice with sepsis myocardial injury (Figure 1B). FAM134B and LC3-II/I expression in the myocardial tissue of mice in sham, sepsis, sepsis + Rap, and sepsis + 3-MA groups were evaluated using IHC analysis. The protein expression of FAM134B (green fluorescence) and LC3-II/I (red fluorescence) were significantly increased in the sepsis mice compared with the sham group (Figure 1C). The protein levels of FAM134B and LC3-II/I were increased in the sepsis + Rap group and decreased in the sepsis + 3-MA group, compared with the sepsis mice (Figure 1C).

### FAM134B protects against sepsis myocardial injury in mice

**Figure 2:**
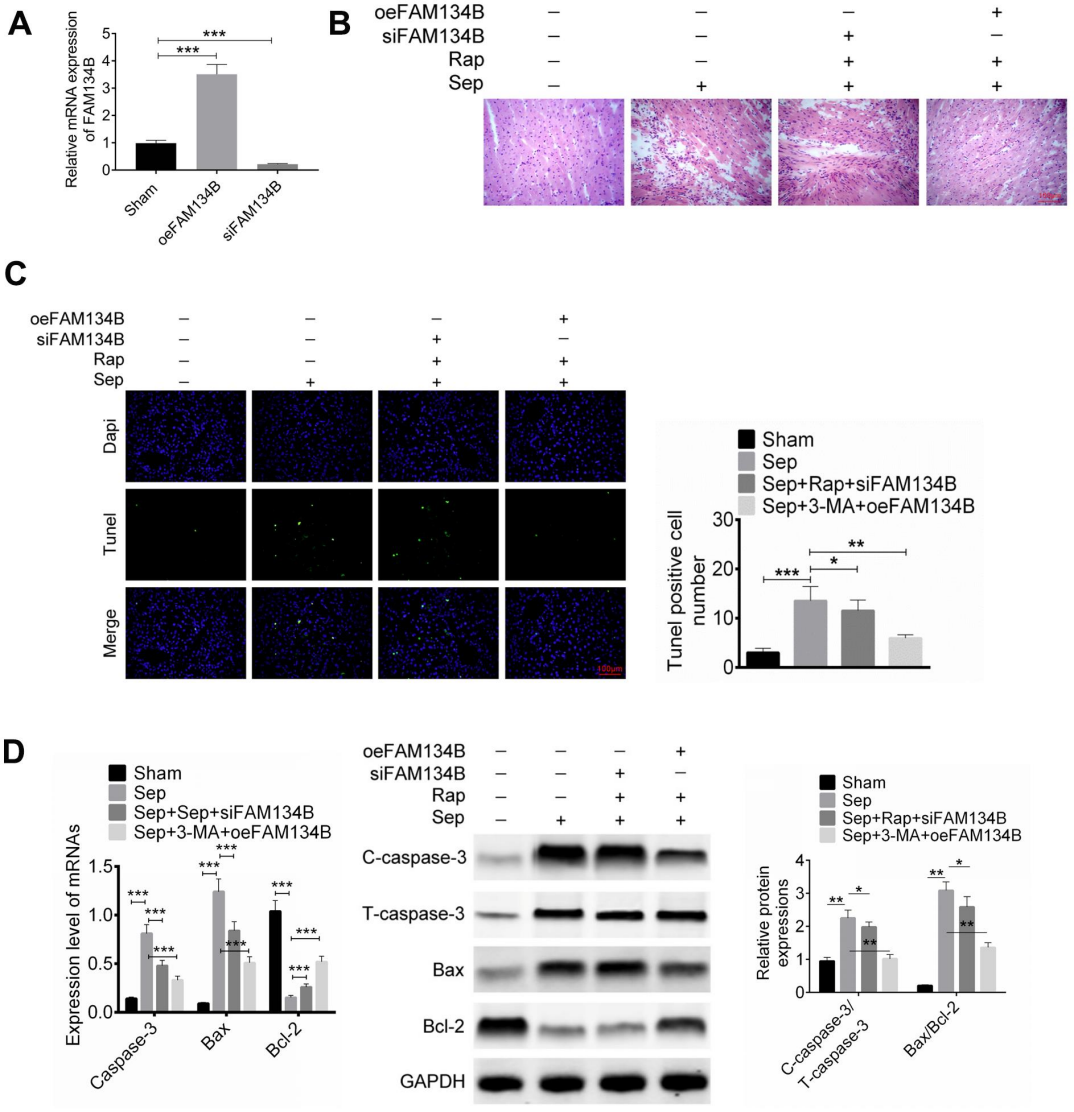
**Effect of FAM134B on sepsis myocardial injury in mice.** Mice were treated with FAM134B knockdown or overexpression and then with cecal ligation and puncture treatment. Mice were divided into four groups: Sham, Sep, Sep+Rap+siFAM134B, and Sep+3-MA+oeFAM134B. (**A**) The expression of FAM134B in myocardial tissue was detected by RT-PCR. (**B**–**D**) Myocardial tissue morphology and apoptosis were then identified by H&E staining and TUNEL analysis, respectively. Data are shown as mean ± SD. *P<0.05, **P<0.01 and ***P<0.001. Sep: Sepsis. SD: standard deviation.

To further identify the effect of FAM134B on sepsis myocardial injury, mice were treated with FAM134B knockdown or overexpression, followed by cecal ligation and puncture treatment. After transfection, the expression of FAM134B in the myocardial tissue of mice was examined using RT-PCR (Figure 2A). Mice were divided into four groups: sham, Sep, Sep + Rap + siFAM134B, and Sep + 3-MA + oeFAM134B. Myocardial tissue morphology and apoptosis were then identified using H&E staining and TUNEL analysis, respectively. As shown in Figure 2A, 2B, injury and apoptosis in the cardiac tissue were observed in the Sep group, and siFAM134B reversed the protective effect of Rap against sepsis myocardial injury and apoptosis in mice. However, the overexpression of FAM134B relieved the sepsis myocardial injury and apoptosis in mice treated with Sep + 3-MA (Figure 2A, 2B). Cell apoptosis-related proteins, including cleaved-caspase-3/caspase-3, Bax, and Bcl-2, were then examined using western blot analysis. Sepsis-induced myocardial injury significantly increased the expression of cleaved-caspase-3 and Bax and decreased the expression of Bcl-2, compared with that in the sham group (Figure 2C). The ratios of cleaved-caspase-3/caspase-3 and Bax/Bcl-2 in the Sep + Rap + siFAM134B-treated mice and Sep + 3-MA + oeFAM134B-treated mice were dramatically decreased compared with those in the Sep group (Figure 2C).

### FAM134B reduces inflammatory response and mediates autophagy-related protein expression of sepsis myocardial injury in mice

**Figure 3:**
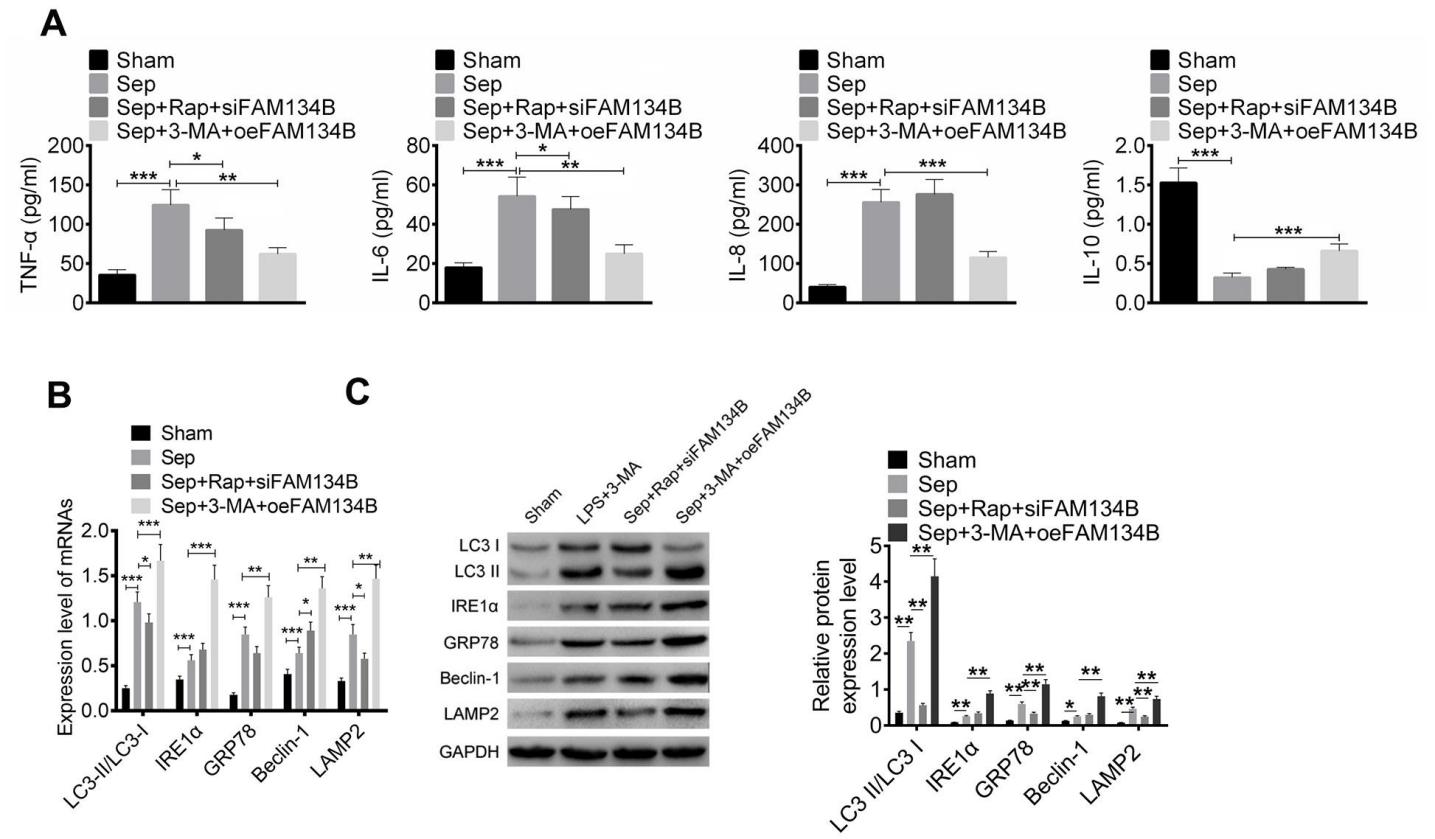
**FAM134B reduced the inflammatory response and regulated the autophagy-related protein expressions of sepsis myocardial injury in mice.** (**A**) The levels of TNF-α, IL-6, IL-8, and IL-10 were evaluated by ELISA. (**B**, **C**) The mRNA and protein expressions of LC3-II/I, IRE1α, GRP78, Beclin-1, and LAMP2 in the myocardial tissues of four groups (Sham, Sep, Sep+Rap+siFAM134B, and Sep+3-MA+oeFAM134B) were detected by RT-PCR and western blot, respectively. Data are shown as mean ± SD. *P<0.05, **P<0.01 and ***P<0.001. Sep: Sepsis. SD: standard deviation.

We then identified inflammation in the mice with sepsis myocardial injury, and the levels of TNF-α, IL-6, IL-8, and IL-10 were evaluated using ELISA. It was found that sepsis-induced myocardial injury significantly promoted the release of TNF-α, IL-6, and IL-8 and decreased the IL-10 level, compared with that in the sham group (Figure 3A). The levels of TNF-α and IL-6 in Sep + Rap + siFAM134B treatment were significantly decreased compared to those in the Sep group. In mice treated with Sep + 3-MA + oeFAM134B, FAM134B overexpression significantly decreased the levels of TNF-α, IL-6, and IL-8, and an increased release of IL-10 was observed in the myocardial tissue, compared with the Sep mice (Figure 3A).

The expression of autophagy-related proteins*,* including LC3-II/I, IRE1α, GRP78, Beclin-1, and LAMP2 in the myocardial tissues of the four groups (sham, Sep, Sep + Rap + siFAM134B, and Sep + 3-MA + oeFAM134B) were detected using RT-PCR and Western blotting. The results showed that the mRNA and protein expression levels of LC3-II/I, IRE1α, GRP78, Beclin-1, and LAMP2 were significantly increased in Sep mice compared with sham mice (Figure 3B, 3C). The overexpression of FAM134B effectively reduced the expression of LC3-II/I, IRE1α, GRP78, Beclin-1, and LAMP2 in the myocardial tissues of mice treated with Sep + 3-MA, compared with the Sep mice (P < 0.01, Figure 3B, 3C). In addition, siFAM134B decreased the expression of LC3-II/I, GRP78, and LAMP2 in the myocardial tissues of mice treated with Sep + Rap.

### FAM134B expression was increased in LPS-treated mouse cardiomyocytes

**Figure 4:**
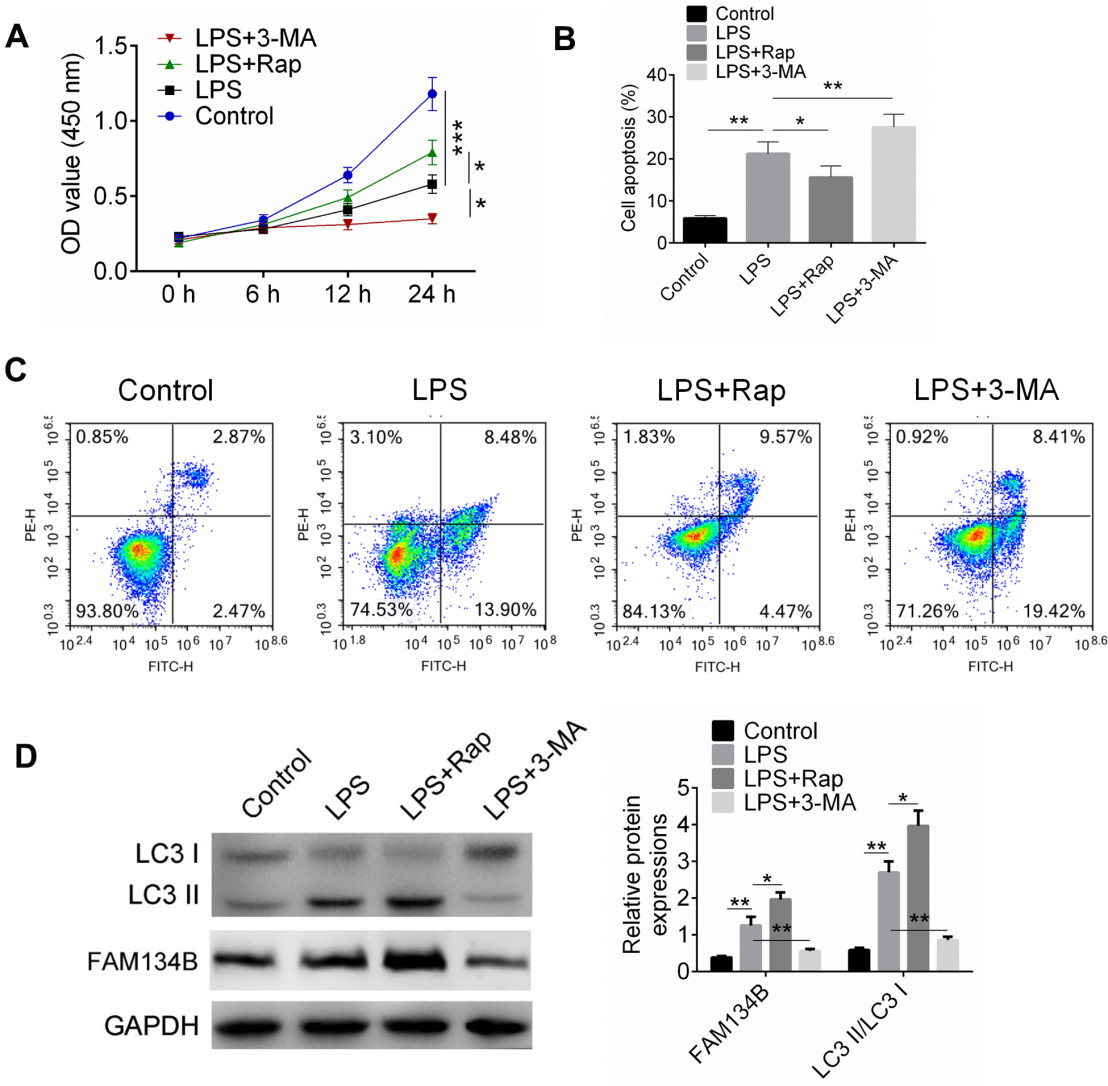
**FAM134B expression was increased in LPS-treated mouse cardiomyocytes.** (**A**) Cardiomyocytes were treated with LPS, or LPS+RAP, or LPS+3-MA. Cell proliferation was examined by CCK8 assay. (**B**, **C**) Cell apoptosis was measured by Annexin V/PI staining. (**D**) The expressions of LC3-II/I and FAM134B were identified by western blot analysis. Data are shown as mean ± SD. *P<0.05, **P<0.01 and ***P<0.001. SD: standard deviation.

We then identified the expression and effect of FAM134B on LPS-induced mouse cardiomyocytes. The cardiomyocytes were treated with LPS, LPS + Rap, or LPS + 3-MA. Cell proliferation and apoptosis were identified using CCK8 and Annexin V/PI staining, respectively. The results showed that LPS treatment significantly inhibited cell viability and induced cell apoptosis (Figure 4A, 4B, P < 0.01). Rap treatment alleviated the injury and cell apoptosis, while 3-MA promoted the effect of LPS treatment (Figure 4A, 4B).

The expression levels of LC3-II/I and FAM134B were examined using western blot analysis (Figure 4C). The data showed that LPS induced the expression of LC3-II/I and FAM134B, while Rap showed a stimulating effect on LC3-II/I and FAM134B expression. Moreover, 3-MA, an autophagy inhibitor, inhibited the expression of LC3-II/I and FAM134B. The results indicated that LPS could induce autophagy in cardiomyocytes, and facilitating autophagy showed a protective effect on LPS-treated cardiomyocytes.

### FAM134B-mediated autophagy reduces cell apoptosis and inflammation response in LPS-treated mouse cardiomyocytes

**Figure 5:**
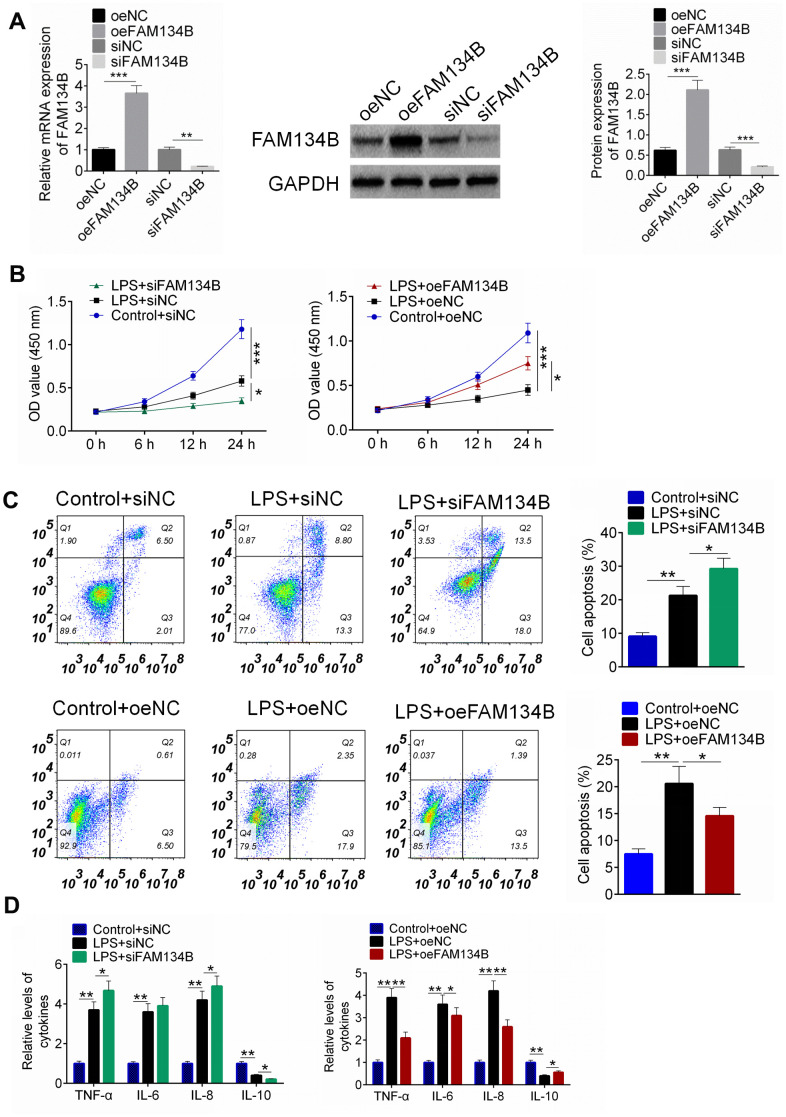
**The effect of FAM134B on LPS-treated mouse cardiomyocytes.** (**A**) Cardiomyocytes were transfected with oeNC, oeFAM143B, siNC, siFAM134B, and the mRNA and protein expressions of FAM134B were detected by RT-PCR and western blot analysis. (**B**) Cardiomyocytes with transfection were then treated with LPS, and cell proliferation was identified by CCK8 assay. (**C**) Cell apoptosis was examined by Annexin V/PI staining. (**D**) The release of inflammatory factors was evaluated by ELISA. Data are shown as mean ± SD. *P<0.05, **P<0.01 and ***P<0.001. SD: standard deviation.

To verify that FAM134B overexpression showed a protective effect on LPS-treated mouse cardiomyocytes, cardiomyocytes were transfected with siFAM134B or oeFAM134B, followed by LPS treatment. Cardiomyocytes were divided into six groups: Control + oeNC, LPS + oeNC, LPS + oeFAM134B, control + siNC, LPS + siNC, and LPS + siFAM134B. As shown in Figure 5A, transfection was examined using RT-PCR and western blot analysis. Cell proliferation, apoptosis, and inflammatory cytokines were subsequently identified. It was found that LPS inhibited cell proliferation, induced cell apoptosis, and stimulated the inflammatory response of cardiomyocytes (Figure 5B–5D, P < 0.001). However, siFAM134B aggravated the injury of LPS treatment on cardiomyocytes, and the oeFAM134B transfection alleviated the LPS-induced injury of LPS treatment on cardiomyocytes (Figure 5B–5D, P < 0.05).

**Figure 6:**
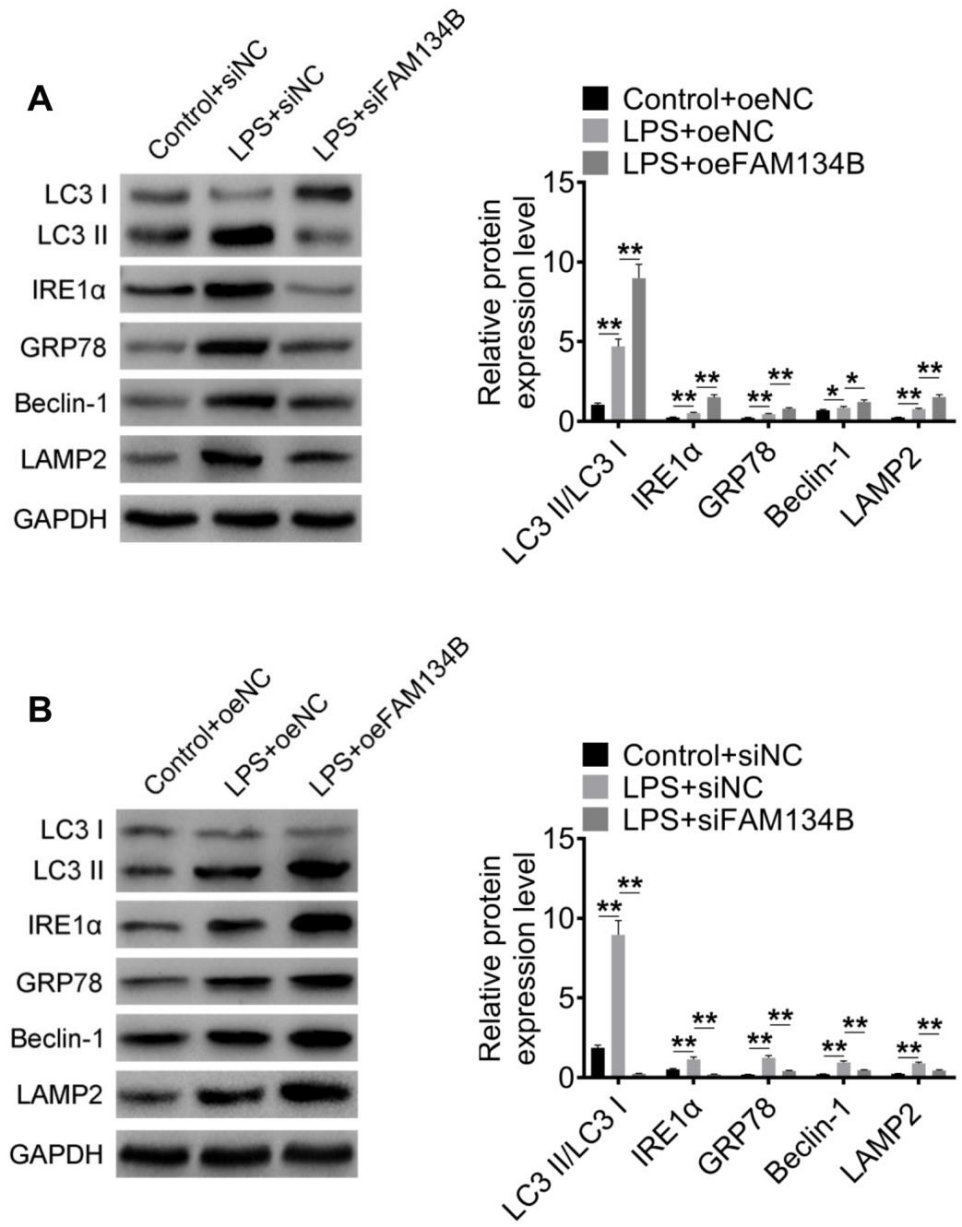
**The effect of FAM134B on the expressions of autophagy-related proteins.** (**A**, **B**) Cardiomyocytes were transfected with oeNC, oeFAM143B, siNC, siFAM134B, and then treated with LPS. The protein expressions of LC3-II/I, IRE1α, GRP78, Beclin-1, and LAMP2 were detected by western blot analysis. Data are shown as mean ± SD. *P<0.05, **P<0.01 and ***P<0.001. SD: standard deviation.

The expression of autophagy-related proteins was examined using western blot analysis. The results revealed that LPS treatment promoted the expression of LC3-II/I, IRE1α, GRP78, Beclin-1, and LAMP2, and the knockdown of FAM134B suppressed the expression of autophagy-related proteins (Figure 6A, P < 0.05). Furthermore, the over-expression of FAM134B significantly promoted the expression of autophagy-related proteins, compared with the LPS-treated cardiomyocytes (Figure 6B, P < 0.05). The results showed that FAM134B overexpression induced autophagy, which showed a protective effect on LPS-treated mouse cardiomyocytes.

## DISCUSSION

Autophagy is an important way to maintain homeostasis of the internal environment of the body. It can engulf, encapsulate, and degrade its own cytoplasmic proteins or organelles, providing raw materials for the normal survival and metabolism of cells [7]. When the function of endoplasmic reticulum changes, cells activate selective autophagy to clear the damaged endoplasmic reticulum or endoplasmic reticulum fragments. The main function of ER autophagy is to improve the intracellular environment and protect cell activity [6]. Previous studies reported that FAM134B plays a crucial role in ER autophagy, and it is an ER-anchored autophagy receptor that mediates ER delivery into the lysosomes through sequestration into autophagosomes [11]. In the present study, our goal was to explore the effect of FAM134B-mediated ER autophagy in sepsis myocardial injury in mice. We first constructed a septic mouse model cecal ligation and puncture assay. The treatment using Rap, an autophagy inducer [18], has been reported to induce autophagy, decrease the activation of inflammasomes, and alleviate pathological injuries during the acute stage of sepsis in CLP mice, in a preliminary study [19]. Our results indicate that the activation of autophagy by Rap effectively decreases the damage induced by sepsis in the myocardial tissue. Moreover, 3-MA is an autophagy inhibitor [20, 21] that aggravates myocardial tissue injury in mice. Furthermore, we found that the expression of LC3-II/LC3-I and FAM134B increased in the Sep + Rap-treated group, which might indicate that FAM134B induced autophagy had a protective effect against sepsis myocardial injury.

We subsequently studied the effect of FAM134B knockdown or overexpression on the apoptosis and inflammatory response in CLP mice. Mice were transfected with siFAM134B and FAM134B overexpression, followed by CLP and Rap/3-MA treatment. Interestingly, we found that FAM134B overexpression could alleviate myocardial injury and apoptosis in the Sep + 3-MA group. The expression of cleaved-caspase-3 and Bax/Bcl-2 was also regulated by FAM134B overexpression in the Sep + 3-MA group. In the Sep + Rap + siFAM134B group, myocardial injury and apoptosis were aggravated. The results showed that FAM134B could induce autophagy in mice with sepsis myocardial injury and, therefore, protect the myocardial tissue against sepsis-induced injury. The effect of oeFAM134B on Sep + Rap mice and the effect of siFAM134b on Sep + 3-MA mice was not investigated in our study. This is a limitation of our study and should be part of future research work. Sepsis is a systemic inflammatory response syndrome caused by infection [22, 23]. The release of inflammatory cytokines plays a crucial role in the process of sepsis myocardial injury [24]. A number of studies have reported that an excessive productions of TNF-α, IL-6, and IL-8 severely enhances myocardial dysfunction during sepsis [25, 26]. Mitochondria are abundant in the heart, not only involved in energy supply, but also involved in the regulation of intracellular calcium [27]. Therefore, the degree of mitochondrial dysfunction is closely related to cardiac dysfunction. Inflammatory factors such as TNF-α, IL-6, and IL-8 can cause mitochondrial dysfunction and aggravate myocardial injury [28]. IL-10 is a well-known anti-inflammatory factor that induces an immune repressor pathway in sepsis [29]. In our study, FAM134B overexpression reduced the productions of TNF-α, IL-6, and IL-8, and enhanced the release of IL-10. The results illustrated that FAM134B mediated the inflammatory reaction and alleviated myocardial dysfunction. To further identify if the upregulation of FAM134B activated the process of autophagy, the expressions of LC3-II, IRE1α, GRP78, Beclin-1, and LAMP2 were evaluated. These proteins are key factors in autophagy. The present study showed that FAM134B overexpression promoted the expressions of LC3-II, IRE1α, GRP78, Beclin-1, and LAMP2. Moreover, we examined the effect of FAM134B on LPS-induced cardiomyocytes, and the results showed that FAM134B promoted autophagy, which showed a protective effect on LPS-treated mouse cardiomyocytes.

In summary, FAM134B-mediated ER autophagy showed a protective effect against sepsis myocardial injury in mice by reducing inflammation and tissue apoptosis, which may provide new insights for sepsis myocardial injury therapies.

### Availability of data and materials

All data from this study are available on a reasonable request in this published article.
